# Emerging Cross-Resistance to Cefiderocol and Ceftazidime-Avibactam in KPC-Producing *Klebsiella pneumoniae* During Ceftazidime-Avibactam Therapy

**DOI:** 10.3390/antibiotics15070701

**Published:** 2026-07-17

**Authors:** Cristina Riazzo, Cristina Elías-López, Montserrat Muñoz-Rosa, Cristina Arjona-Torres, Tania Blanco-Martín, Isabel Machuca, Julian Torre-Cisneros, Irene Gracia-Ahufinger, Nicolas Kieffer, Jorge Arca-Suárez, Luis Martínez-Martínez

**Affiliations:** 1 Maimonides Biomedical Research Institute of Cordoba, Reina Sofía University Hospital, University of Cordoba (IMIBIC/HURS/UCO), 14004 Cordoba, Spain; 2 Unit of Microbiology, Reina Sofía University Hospital, 14004 Cordoba, Spain; 3 CIBER de Enfermedades Infecciosas (CIBERINFEC), Instituto de Salud Carlos III, 28029 Madrid, Spain; 4 Servicio de Microbiología Clínica and Grupo de Investigación en Microbiología, Instituto de Investigación Biomédica de A Coruña (INIBIC), Complexo Hospitalario Universitario de A Coruña (CHUAC), SERGAS, Universidade da Coruña (UDC), 15006 A Coruña, Spain; 5 Infectious Diseases Service, Reina Sofía University Hospital, 14004 Cordoba, Spain; 6 Department of Agricultural Chemistry, Soil Sciences and Microbiology, University of Cordoba, 14071 Cordoba, Spain

**Keywords:** *Klebsiella pneumoniae*, KPC, ceftazidime/avibactam, cefiderocol

## Abstract

**Background/Objectives:** Ceftazidime-avibactam (CAZ/AVI) and cefiderocol (FDC) retain activity against most clinical isolates of KPC-producing *Klebsiella pneumoniae* (KPC-Kp). Nevertheless, the emergence of cross-resistance between both agents has become an increasing clinical concern. In this study, the development of resistance to both CAZ/AVI and FDC was retrospectively investigated in KPC-Kp isolates recovered from patients treated with CAZ/AVI between 2014 and 2023. **Methods:** Twenty-two isolates (pre- and post-therapy) obtained from nine patients were included. Demographic and clinical data were collected. FDC susceptibility testing was determined for both clinical isolates and *E. coli* TOP10 transformants by reference broth microdilution using iron-depleted medium, and MICs were interpreted according to EUCAST breakpoints. Resistance mechanisms were characterized using whole genome sequencing. **Results:** Cross-resistance to both CAZ/AVI and FDC emerged in six patients (66.7%) during CAZ/AVI therapy, after a median treatment duration of 12.8 ± 7.6 days. The presence of different KPC-3 variants in the CAZ/AVI-FDC resistant isolates was revealed by genomic analysis. Furthermore, the role of KPC-28, KPC-31, KPC-47, KPC-94, KPC-95, and KPC-148 in mediating reduced susceptibility to CAZ/AVI and FDC was confirmed by AST of transformants carrying *bla*_KPC_ variants. However, differences in MIC values compared with those obtained for the corresponding clinical isolates supported the relevance of additional mechanisms of co-resistance. The presence of ferric citrate transport (FEC) system was also associated with higher FDC MICs. Mutations in genes coding siderophore-iron transporter, PBPs, sensor histidine kinase or Tol-Pal system were also found in some of the isolates. **Conclusions:** These findings highlight the ability of KPC-Kp to develop co-resistance to both CAZ/AVI and FDC during CAZ/AVI therapy.

## 1. Introduction

Carbapenem-resistant *Klebsiella pneumoniae* has emerged as a critical global concern among multidrug-resistant Gram-negative bacteria, mainly due to the extensive spread of *K. pneumoniae* isolates producing diverse carbapenemases, including KPC enzymes. Despite the increasing dissemination of metallo-β-lactamases or OXA-48 enzymes, KPC enzymes are arguably among the most prevalent carbapenemases worldwide [1,2,3,4].

The introduction of ceftazidime/avibactam (CAZ/AVI) represented a breakthrough in the management of infections caused by KPC-producing Enterobacterales [5,6,7]. Nevertheless, the growing use of this combination has been accompanied by the emergence of CAZ/AVI-resistant KPC-producing *Klebsiella pneumoniae* (KPC-Kp) strains [8,9,10,11,12].

Cefiderocol (FDC) is a siderophore cephalosporin highly active against carbapenem-resistant Enterobacterales, including KPC-Kp. It has been described as a “Trojan horse” entering bacterial cells using iron transport systems [11,12]. Although the ceftazidime- and cefepime-related side chains on its structure provide improved hydrolytic stability against β-lactamases, several studies [13,14] have indicated that resistance mechanisms selected during CAZ/AVI exposure (particularly KPC Ω-loop mutations) may confer cross-resistance to FDC. Indeed, it has been shown that the D179Y substitution in variants such as KPC-31 and KPC-33 is responsible for the formation of a long-lived covalent intermediate leading to a decreased and rate-limiting deacylation step which affects both CAZ/AVI and FDC [15].

In addition, alterations in genes involved in iron transport across the cell membrane, particularly *cirA* and *fiu*, have been reported to affect FDC susceptibility in *K. pneumoniae* [16,17,18]. A previous study further demonstrated that the combination of mutations in the *bla*_KPC_ allele and *envZ* was sufficient to produce a ≥256-fold increase in the FDC MIC [16]. Recently, the presence of a plasmid-borne ferric citrate transport (FEC) system in some KPC-Kp strains has been demonstrated to be associated with reduced susceptibility to FDC, as it causes transcriptional repression of *fiu*, *cirA*, *fepA*, and *fhuA* siderophore receptor genes under ferric citrate exposure [17].

The emergence of CAZ/AVI-resistant KPC-Kp harboring different KPC-variants in patients treated with this combination has been previously described by our group [10,18]. However, the activity of FDC against these isolates and the potential for cross-resistance have not been investigated. After these reports, additional cases of CAZ/AVI-resistant KPC-Kp have also been identified in our center.

The aim of this study was to investigate the emergence of cross-resistance to CAZ/AVI and FDC among KPC-Kp isolates selected during CAZ/AVI therapy, and to characterize the mechanisms associated with reduced FDC susceptibility.

## 2. Results

### 2.1. Patient Data and Phenotypic Characterization of KPC-Producing K. pneumoniae

Demographic data, clinical presentation, outcome, treatment duration of the patients with CAZ/AVI and antimicrobial susceptibility of the isolates included in this study are summarized in Table 1.

All the pre-therapy CAZ/AVI-susceptible isolates (n = 9) were susceptible to FDC with MIC values ranging from 0.12 to 1 mg/L. For 11/13 post-therapy isolates, the MIC increased ≥4-fold (two doubling dilutions) compared to the corresponding parental isolate. This translated into FDC clinical resistance for 9 isolates obtained from 6 of 9 patients (66.7%) with a MIC range of 4 to >32 mg/L (Table 1). Antimicrobial susceptibility to other antibiotics was variable. Notably, all pre- and post-therapy isolates were susceptible to imipenem-relebactam and meropenem-vaborbactam, and only one post-therapy isolate was resistant to aztreonam-avibactam. The median duration of treatment with CAZ/AVI was 12.8 (range 6 to 27 days) before the first CAZ/AVI-FDC resistant isolate emerged. No patients were treated with FDC.

### 2.2. Genomic Characterization

It was revealed by whole genome sequencing that all the pre-therapy isolates susceptible to both CAZ/AVI and FDC contained *bla*_KPC-3_. Post-therapy CAZ/AVI-FDC-resistant isolates (n = 13) contained different variants of KPC-3: KPC-28, KPC-31 (n = 4), KPC-39, KPC-47, KPC-48, KPC-85, KPC-94 or KPC-95. Additional acquired resistance genes for β-lactams and other antibiotics are presented in Table 2. Furthermore, all isolates expressed the beta-lactamases TEM-1 and SHV-11. Initially, ResFinder detected that all isolates carried the OXA-9 beta-lactamase, but this was not detected by CARD; manual sequence analysis revealed that the OXA-9 was truncated.

All 22 isolates presented an insertion of one nucleotide in the *ompK35* gene leading to a premature stop codon at position 67, resulting in a truncated protein and a two-amino-acid insertion (GD) at position 115 of the Ompk36 protein.

Different mutations in genes, which are not primarily known to be a direct cause of FDC resistance (Table S1), were identified by variant calling analysis. Notably, the KPC-39-producing strain (CHURS_183111) exhibited a non-synonymous mutation leading to an amino acid change (P266L) in a gene that was annotated by Bakta as *cirA*, which encodes a catecholate-type siderophore receptor. However, subsequent bioinformatics analysis using AlphaFold and Foldseek revealed that this gene should rather be annotated as a TonB-dependent receptor. To the best of our knowledge, no mutation in this gene has been associated with cefiderocol resistance. Furthermore, alterations in the *mrdA* gene were carried by five isolates that led to the H375R (n = 3) and the D354A (n = 2) substitution in PBP-2; two isolates showed an amino acid change (P389S) in the sensor histidine kinase EnvZ; and one isolate carried a mutation in the *tolQ* gene (Tol-Pal system) leading to the E48G substitution.

**Figure 1:**
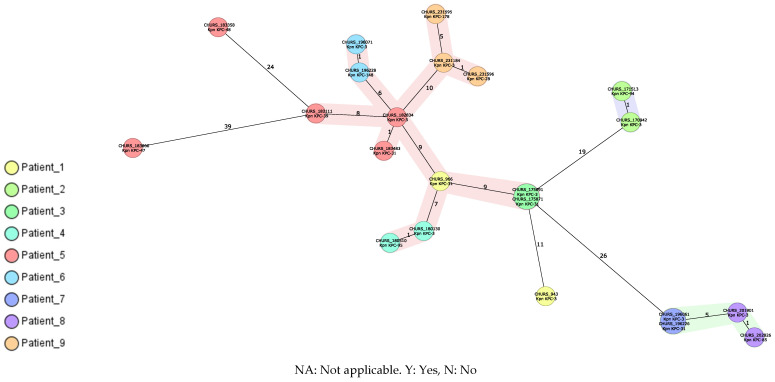
Minimum spanning tree of 22 KPC-producing *K. pneumoniae* isolates indicating the strain number and KPC variant in the circles. Each circle represents one or more isolates, each color represents one patient, and the number of different alleles is indicated on the edges between connected isolates (nodes). Shaded areas indicate cgMLST clusters comprising isolates differing by ≤10 alleles. Distance based on cgMLST of 2358 targets using the parameters “pairwise ignoring missing values”.

All isolates were typed as *K. pneumoniae* ST512. The minimum spanning tree (Figure 1) revealed a major cluster of closely related *K. pneumoniae* isolates (≤10 allelic differences) recovered from different patients over the entire study period. This cluster included isolates carrying different KPC variants within a shared genetic lineage. A range of SNP distances between 0 and 95 was shown by cgSNP analysis of the isolates recovered from individual patients (Table S2).

**Figure 2:**
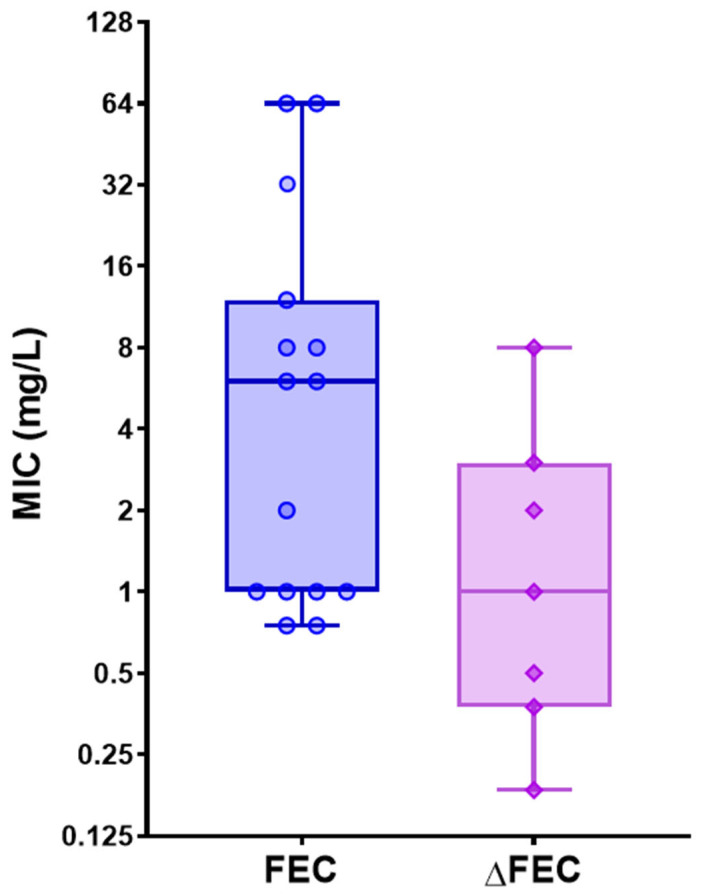
Distribution of FDC MIC values according to the presence of the FEC system.

The FIB(K) replicon and the *fec* operon were found together in 15/22 (68.2%) isolates. In most cases, the presence of both FIB(K) and *fec* gene cluster was concordant among all pre- and post-therapy isolates from the same patient. However, the post-therapy isolate carrying *bla*_KPC-148_ showed the presence of the FIB(K) replicon but lacked the *fec* operon in contrast to its parental, *bla*_KPC-3_, which displayed both. In contrast, both the FIB(K) replicon and the *fec* gene cluster were found in the post-therapy KPC-95-Kp, whereas the parental KPC-3-Kp did not present any of them. Isolates carrying the *fec* operon exhibited higher FDC MIC values than isolates lacking the operon. Median FDC MICs were 6 mg/L (range, 0.75–64 mg/L) and 1 mg/L (range, 0.18–8 mg/L), respectively (Figure 2).

### 2.3. Susceptibility Testing of E. coli Transformants Producing KPC Variants

Compared to *E. coli* (pKPC-3), FDC MICs were ≥4-fold (two doubling dilutions) higher in 6 out of 10 transformants, namely, *E. coli* harboring pKPC-28, pKPC-31, pKPC-47, pKPC-94, pKPC-95, and pKPC-148 (Table 3). Transformants with the highest CAZ/AVI MICs (>256 mg/L) consistently exhibited the highest FDC MICs (1–4 mg/L), whereas those with lower CAZ/AVI MICs (2–8 mg/L) retained lower FDC MICs (0.06–0.5 mg/L). Amino acid changes associated with increased FDC MICs were distributed across different regions of the KPC protein, suggesting that reduced FDC susceptibility may not be exclusively linked to alterations within the Ω-loop. Variants carrying D179Y and the deletion of 242-243_GT exhibited the largest increase in FDC MIC (Table 3).

## 3. Discussion

Although FDC has emerged as a valuable therapeutic option for the treatment of infections produced by carbapenem-resistant Gram-negative bacteria, previous studies have shown that *K. pneumoniae* isolates producing KPC variants associated with CAZ/AVI resistance may exhibit increased MICs of/resistance to FDC [14].

In the present study, the emergence of concomitant resistance to CAZ/AVI and FDC is described in clinical isolates of KPC-Kp recovered after CAZ/AVI treatment. The endemic circulation of KPC-Kp at our institution for more than 14 years has provided a unique epidemiological setting to investigate the evolution of resistance under antimicrobial selective pressure. Notably, most isolates of this study were recovered before FDC became available at our center in early 2020 through the Special Access Scheme. Therefore, the emergence of reduced FDC susceptibility in the majority of cases cannot be explained by direct FDC exposure, supporting that resistance may arise as a collateral consequence of CAZ/AVI-driven selection of KPC-Kp.

Recently, clinical emergence of FDC cross-resistance among CAZ/AVI resistant KPC-producing *K. pneumoniae* has also been reported by other investigators [19,20,21]. These observations are extended by the present findings, in which functional evidence for the contribution of new KPC variants is provided, and additional factors potentially associated with reduced FDC susceptibility are identified. The contribution of KPC-28, KPC-31, KPC-47, KPC-94, KPC-95, and KPC-148 to reduced FDC susceptibility was confirmed by functional characterization of recombinant clones, supporting a direct role of specific KPC variants in cross-resistance. In contrast, KPC-39, KPC-48, KPC-85 and KPC-178 produced in *E. coli* did not have a clear impact on FDC MIC. Consistent with previous reports, variants carrying the D179Y substitution produced the largest increases in FDC MICs, including those with additional amino acid substitutions. However, not all KPC variants with alterations within the Ω-loop were associated with increased FDC MICs, indicating that the impact of individual substitutions is highly mutation-specific, depending on the amino acid change. Notably, D179Y-containing KPC variants, particularly KPC-31, have been increasingly reported worldwide following ceftazidime-avibactam therapy and are among the best-characterized mechanisms of acquired ceftazidime-avibactam resistance and treatment failure [22]. In contrast, several of the other variants identified in our collection remain uncommon, and their epidemiological distribution and clinical significance require further investigation.

Remarkably, increases in MICs of FDC observed in *E. coli* transformants were substantially lower than those observed in the corresponding clinical isolates. Although this comparison should be interpreted with caution, as the genetic backgrounds are not identical, this difference may reflect that KPC variants alone are unlikely to fully explain the resistant phenotype and supports the contribution of additional resistance determinants.

The twenty-two studied clinical isolates share the same truncating OmpK35 mutation and GD insertion in OmpK36. As these porin defects are conserved among all isolates, they do not explain the observed MIC variability in the isolates by themselves; however, the decreased outer membrane permeability caused by the simultaneous alteration in the two major porins of *K. pneumoniae* contributes synergistically with KPC variants and iron uptake pathways to the observed resistance phenotype [23,24].

In agreement with this hypothesis, a significant association between the presence of the *fec* operon and higher FDC MIC values was identified. The FEC system has been previously linked to reduced FDC susceptibility through repression of siderophore receptors involved in FDC uptake [17]. This is exemplified in the post-therapy isolate producing KPC-95 (with the D179Y substitution) displaying one of the highest FDC MICs observed in this study, and much higher than the MIC for the corresponding transformant *E. coli* p (KPC-95). This post-therapy isolate harbored the *fec* operon, in contrast to the corresponding pre-therapy isolate, which suggests that alterations in iron uptake may act synergistically with an altered KPC hydrolytic activity to further increase FDC resistance.

Conversely, although a 4-fold increase in FDC MIC values for *E. coli* (pKPC-148) with respect to *E. coli* (pKPC-3) was observed, there was not a clear impact on the corresponding clinical isolate. Notably, this isolate lacked the *fec* operon despite retaining the IncFIB (K) replicon, pointing to the possibility of partial plasmid rearrangements or deletion events affecting the structure of this operon. Furthermore, the KPC-148-associated insertion (Ins275_EAVYTRAPNKDDKYS) was present in only approximately 60% of sequencing reads (167 reads), suggesting potential heterogeneity within the bacterial population that may have attenuated the level of FDC resistance.

Additional mutations affecting iron acquisition and cell wall synthesis were also detected. In particular, the KPC-39-producing isolate harbored two missense mutations resulting in amino acid substitutions in a protein related to a TonB-dependent receptor (P266L) and PBP-2 (H375R). Although expression of KPC-39 alone did not increase FDC MICs in recombinant clones, these additional alterations may have contributed to the increased MIC observed in the clinical post-therapy isolate. Also, the presence of the P389S change in the histidine kinase EnvZ together with the H375R change in PBP-2 of two isolates from patient 5 (CHURS_183358 and CHURS_183360) harboring KPC-47 and KPC-48 could also have contributed to the high level of FDC resistance in those clinical isolates. A missense mutation resulting in a V147G change previously identified in the sensor histidine kinase EnvZ has been demonstrated to be responsible for FDC MIC increase when combined with *bla*_KPC-121_ [16]. Although a different amino acid substitution was found in our isolates, this change could be involved as an additional factor contributing to FDC resistance. On the other hand, mutations in the *mrdA* gene have been previously described in KPC-Kp [25] with concomitant resistance to CAZ/AVI and FDC; however, their specific contribution to resistance remains unknown, as experimental evidence has not been provided yet. Additional studies are warranted to evaluate this possibility.

These results indicated that FDC susceptibility cannot be assumed in CAZ/AVI-resistant KPC-producing *K. pneumoniae* and reinforce the need for continuous susceptibility monitoring during therapy.

cgMLST analysis revealed that isolates from six patients recovered over the 9 year-period of study clustered within a threshold of ≤10 allelic differences, suggesting the persistence of a closely related clonal lineage. The observation of resistance emergence across multiple patients within the same genetic background may indicate repeated adaptive evolution within a persistent hospital-associated clone. The analysis of cgSNP showed that, in most cases, sequential isolates were closely related (≤25 SNPs) (Table S2). However, two isolates (CHURS_183358 and CHURS_183360) of patient 5 exhibited SNP distances (45 and 95) too high to be considered derived directly from the pre-therapy isolate.

Patient 5 represented the most remarkable example of within-host diversification, with the sequential emergence of four distinct KPC variants (KPC-39, KPC-47, KPC-48 and KPC-31) following ceftazidime-avibactam exposure. This observation illustrates the remarkable adaptive potential of KPC-producing *K. pneumoniae* under sustained antimicrobial selective pressure. Notably, three isolates in this patient (CHURS_183358, CHURS_183360 and CHURS_183111) carried a mutation in the *mutS* gene, resulting in the same amino acid substitution (T115P). This finding raises the possibility that they represent hypermutator strains, which could explain the elevated SNP accumulation and supports their consideration as members of the same clonal lineage rather than unrelated isolates [26].

In the present series, two patients died; however, both had highly complex underlying clinical conditions, and no direct association between the emergence of this resistance phenotype and mortality could be established. One of these patients was switched to meropenem-vaborbactam, to which the corresponding isolate remained susceptible; however, treatment was unsuccessful due to the patient’s severe underlying clinical condition. Although imipenem-relebactam, meropenem-vaborbactam and aztreonam-avibactam still showed activity for most isolates, the emerging expansion of isolates co-producing KPC and metallo-β-lactamases such as NDM [27,28] could limit these treatment options. In this scenario, aztreonam-avibactam would remain, for the moment, the only β-lactam therapeutic option. Unfortunately, the identification of a KPC-94-producing *K. pneumoniae* isolate resistant to aztreonam-avibactam further narrows the available therapeutic arsenal.

Practical implications for antimicrobial stewardship are raised by these findings. Current guidance for KPC-producing *Enterobacterales* generally prioritizes CAZ/AVI, meropenem-vaborbactam or imipenem-relebactam when active in vitro, while FDC is usually considered an alternative option [29]. In this context, emergence of CAZ/AVI resistance during therapy should prompt repeat susceptibility testing, including FDC and other β-lactam/β-lactamase inhibitor combinations. These results suggest that FDC should not be assumed to remain active after CAZ/AVI failure, even in patients not previously exposed to FDC.

In the present series, meropenem-vaborbactam and imipenem-relebactam retained activity against most post-therapy isolates, supporting their consideration as alternative options after CAZ/AVI resistance emerges, guided by updated MICs, infection source, previous antimicrobial exposure and local epidemiology. Aztreonam-avibactam may be useful in selected cases, particularly when metallo-β-lactamase co-production is suspected, although susceptibility testing remains essential [30].

Several limitations should be acknowledged. First, the number of isolates carrying individual KPC variants was limited, which restricted the ability to determine variant-specific effects accurately. Second, several sequential isolates were recovered from different anatomical sites. Although cgMLST and cgSNP analyses supported the close genetic relatedness of these isolates, it cannot be completely excluded that differences in the anatomical niche influenced bacterial population dynamics and the selection of resistance mechanisms. Third, functional validation was only performed for KPC enzymes, whereas the contribution of additional alterations identified in cell-wall synthesis genes and others remains inferential. Finally, expression analyses were not performed, preventing assessment of the regulatory impact of the FEC system on FDC susceptibility.

## 4. Conclusions

Selective pressure during therapy with CAZ/AVI for patients with infections caused by KPC-3-producing *K. pneumoniae* may result in resistance to both CAZ/AVI and FDC, even though the latter agent has not been administered. Co-resistance appears to be associated with specific KPC variants and the expression of additional resistance mechanisms. The importance of longitudinal monitoring during CAZ/AVI therapy is underscored by these findings, including the need for repeat susceptibility testing for FDC and other β-lactam/β-lactamase inhibitor combinations to promptly detect emerging resistance and guide appropriate antimicrobial therapy.

## 5. Materials and Methods

### 5.1. Patient Characteristics, Bacterial Isolates and Microbiological Characterization

A collection of 22 isolates of KPC-Kp recovered from 9 patients admitted to the Reina Sofía University Hospital in Cordoba, Spain, between 2014 and 2023, were evaluated. Some of these isolates have been previously characterized [10,18].

The included patients were those previously colonized or infected with KPC-3-Kp (and susceptible to CAZ/AVI) from whom CAZ/AVI resistant isolates were obtained after treatment with this combination. From each patient, the first CAZ/AVI-susceptible KPC-Kp strain obtained from a clinical sample before treatment (pre-therapy) and the first CAZ/AVI-resistant isolate recovered during or after treatment (post-therapy) were selected. Post-therapy CAZ/AVI-resistant *K. pneumoniae* isolates producing a different KPC variant were also included.

Demographic data, clinical presentation and outcome, CAZ/AVI antimicrobial therapy, microbiological samples and date of sampling were recorded.

Microbiological identification was performed by MALDI-TOF/MS (Bruker, Germany). Antimicrobial susceptibility testing (AST) of the 22 clinical isolates was initially performed using both the MicroScan WalkAway system (Beckman Coulter, Madrid, Spain) and EUMDROXF Sensititre panels (ThermoFischer Scientific, Waltham, MA, USA). Carbapenemase detection was investigated by the modified carbapenem inactivation method (mCIM) [10] and the NG-Test CARBA 5 immunochromatography assay (Biotech, Paris, France).

FDC (MedChemExpress, Monmouth Junction, NJ, USA) susceptibility testing was performed in duplicate by standardized microdilution using iron-depleted cation-adjusted Mueller–Hinton broth according to EUCAST guidelines.

*Escherichia coli* ATCC 25922 and *Pseudomonas aeruginosa* ATCC 27853 were used as control strains. MIC values were interpreted using EUCAST breakpoints (v15.0).

### 5.2. Whole Genome Sequencing and Bioinformatic Analysis

Whole genome sequencing with short reads was performed for the 22 selected isolates. DNA was extracted using an automatic MagCore^®^HF16 Plus System with the MagCore^®^Genomic DNA Bacterial Kit 502 (RBC Bioscience, Taipei, Taiwan). Genomic DNA paired-end libraries were generated using an Illumina DNA Prep Kit (Illumina Inc., San Diego, CA, USA). The libraries were sequenced using an Illumina NextSeq 500 sequencer system with 2 × 150 bp paired-end reads (Illumina Inc., USA).

The quality of the raw data was assessed by FastQC on the Galaxy server “https://usegalaxy.eu/ (accessed on 6 February 2025)”. After quality trimming, short reads were subsequently assembled de novo using CLC Genomics Workbench v26.0.2 (Qiagen, Stockach, Germany) and annotated with Bakta v5.0. Variant detection between post-therapy and pre-therapy isolates was performed and manually inspected for confirmation on CLC Genomics Workbench, focusing on previously described genes associated with FDC resistance (e.g., *cirA*, *fiu*, *tonB*, *exbB*, *exbD*, *fecA*, *fbpA*, *efeo*, *piuDC*, *piuA*, *pirR*, *pirA*, *envZ*, *tolQ*, *mrdA*) [16,25,31]. Protein bioinformatics analysis was performed by AlphaFold [32] and Foldseek [33].

Acquired antimicrobial resistance genes were detected using ResFinder v4.7.2 and the Comprehensive Antibiotic Resistance Database (CARD). The sequences of porin genes *ompK35* and *ompK36* were analyzed in silico and compared with those from *K. pneumoniae* ATCC 13883 (NCBI ID: NZ_KN046818.1) using the NCBI BLAST web server “https://blast.ncbi.nlm.nih.gov/Blast.cgi (accessed on 10 March 2026)”. In addition, the FIB (K) replicon and *fec* operon were screened using PlasmidFinder v2.1 and the BLASTN tool, respectively, as previously described elsewhere [17].

Sequence types (STs) were assigned by multilocus sequence typing (MLST) using the scheme of SeqSphere + v11.1.0 (Ridom, Münster, Germany). Genomic relatedness was assessed using a gene-by-gene approach based on core-genome MLST (cgMLST) comprising 2538 targets provided by SeqSphere + v11.1.0 (Ridom, Germany), and the data were visualized in a minimum spanning tree with the “pairwise ignore missing values” parameter. A threshold of ≤10 loci was used for clustering. cgSNP and phylogenetic analyses were performed using CSIPhylogeny v1.4.

### 5.3. Association Between fec Operon and FDC MIC

FDC MIC was determined in duplicate for each isolate, and the mean of the two determinations was used as the representative MIC, as the duplicate measurements did not differ by more than one 2-fold dilution. Isolates were stratified according to the presence or absence of the *fec* operon, and FDC MIC values were summarized descriptively using the median and range. MIC distributions were plotted as box-and-whisker plots including the values for each isolate. No inferential statistical analyses were performed, as serial strains from the same patient were not independent.

### 5.4. Cloning of bla_KPC_ Allelic Variants and Susceptibility Testing of Recombinant Clones

To establish the role of specific KPC alleles in FDC resistance, the 10 *bla*_KPC_ allelic variants found in this study and the reference *bla*_KPC-3_ were cloned. *bla*_KPC_ genes were amplified using primers Kpc-rbs (5′-CTCCACCTTCAAACAAGGAAT-3′) and Kpc-rev (5′-ATCTGCAGAATTCGCCCTTCGCCATCGTCAGTGCTCTAC-3′) as previously described [34]. The amplicons were cloned into the pCR-Blunt II-Topo and electroporated into the *E. coli* TOP10 strain (Invitrogen) resulting in *E. coli* (pKPC-3), *E. coli* (pKPC-28), *E. coli* (pKPC-31), *E. coli* (pKPC-39), *E. coli* (pKPC-47), *E. coli* (pKPC-48), *E. coli* (pKPC-85), *E. coli* (pKPC-94) and *E. coli* (pKPC-95), *E. coli* (pKPC-148) and *E. coli* (pKPC-178). Selection of transformants was carried out using 50 mg/L of kanamycin. Sanger sequencing with T7 promoter and T7 terminator was used for confirmation.

Antimicrobial susceptibility testing of transformants for ceftazidime and ceftazidime-avibactam was determined by gradient strips (BioMérieux, Craponne, France). For FDC, MICs were performed in duplicate using standardized microdilution with iron-depleted cation-adjusted Mueller–Hinton broth prepared according to EUCAST guidelines.

## Figures and Tables

**Table 1:** Overview of demographic and clinical presentation and outcome, treatment duration with CAZ/AVI and antimicrobial susceptibility of clinical isolates included in this study.

Patient	Sex	Age	Clinical Presentation	Unit	CAZ/AVI ^a^ (Days Pre-Culture)	Outcome	Isolate Number	Date	Sample	Therapy Condition	KPC Variant	MIC (mg/L)							
												CAZ/ AVI	FDC 1–2 ^b^	IMP	IMR	MEM	MEV	AZT	AZA
1	F	71	Septic shock of abdominal origin	ICU-General surgery	10	Recovery	CHURS_943	1 September 2014	B	Pre	KPC-3	2/4	1–1	>8	≤0.06/4	>16	0.5/8	>32	0.5
							CHURS_966	25 September 2014	RS	Post	KPC-31	>16/4	8–8	≤1	≤0.06/4	4	0.5/8	16	1
2	M	70	Intraabdominal infection	General surgery-ICU	27	Recovery	CHURS_170942	10 April 2017	PL	Pre	KPC-3	2/4	1–1	>8	0.25/4	>16	1/8	>32	0.75
							CHURS_171513	8 May 2017	PL	Post	KPC-94	>16/4	16–8	≤1	0.25/4	2	2/8	>32	24
3	M	73	Acute cholangitis	Digestive unit	8	Recovery	CHURS_175691	6 December 2017	B	Pre	KPC-3	2/4	0.5–0.5	>8	0.25/4	>16	0.5/8	>32	0.25
							CHURS_175871	18 December 2017	RS	Post	KPC-31	>16/4	8–8	≤1	0.12/4	2	1/8	8	1
4	M	55	Perianal abscess	General surgery	15	Recovery	CHURS_180130	5 January 2018	WS	Pre	KPC-3	2/4	0.12–0.25	>8	0.5/4	>16	0.5/8	>32	0.75
							CHURS_180510	24 January 2018	RS	Post	KPC-95	>16/4	>32–>32	≤1	1/4	2	2/8	32	1
5	M	46	Febrile syndrome of abdominal origin	General surgery	11	Recovery	CHURS_182834	14 May 2018	RS	Pre	KPC-3	2/4	1–0.5	>8	1/4	>16	0.5/8	>32	0.25
							CHURS_183111	28 May 2018	BA	Post	KPC-39	>16/4	4–8	8	2/4	16	1/8	>32	0.75
							CHURS_183358	6 June 2018	PL	Post	KPC-48	>16/4	>32–32	≤1	0.5/4	2	1/8	8	0.75
							CHURS_183483	12 June 2018	PL	Post	KPC-31	>16/4	8–4	≤1	0.12/4	1	0.5/8	16	0.75
							CHURS_183860	27 June 2018	RS	Post	KPC-47	>16/4	32–32	>8	2/4	>16	8/8	>32	0.38
6	M	79	Surgical site infection	General surgery	5	Recovery	CHURS_196071	6 November 2019	WS	Pre	KPC-3	4/4	1–1	>8	1/4	>16	0.5/8	>32	0.38
							CHURS_196228	16 November 2019	RS	Post	KPC-148	>16/4	1–1	4	0.25/4	>16	1/8	>32	0.75
7	M	62	Septic shock of abdominal origin	Digestive unit-ICU	6	Exitus	CHURS_196061	7 November 2019	RS	Pre	KPC-3	2/4	1–0.5	>8	0.25/4	>16	0.5/8	>32	0.5
							CHURS_196226	14 November 2019	PL	Post	KPC-31	>16/4	8–8	≤1	0.12/4	4	0.5/8	16	1
8	M	60	PA-VAP	ICU	7 + 5 ^#^	Recovery	CHURS_201901	27 April 2020	RS	Pre	KPC-3	8/4	1–1	>8	0.25/4	>16	4/8	>32	0.75
							CHURS_202826	29 June 2020	RS	Post	KPC-85	>16/4	2–2	>8	0.25/4	>16	0.5/8	>32	0.5
9	M	45	Septic shock of abdominal origin	General surgery	22	Exitus	CHURS_231184	18 February 2023	RS	Pre	KPC-3	2/4	0.5–0.25	>8	0.12/4	>16	0.5/8	>32	0.5
							CHURS_231595	13 March 2023	RS	Post	KPC-178	>16/4	2–2	>8	0.25/4	>16	1/8	>32	1
							CHURS_231596	13 March 2023	RS	Post	KPC-28	>16/4	4–2	≤1	0.12/4	1	0.5/8	>32	3

^a^ Days of CAZ/AVI treatment before the isolate resistant to CAZ/AVI was cultured. ^b^ MICs of cefiderocol were determined in duplicate. B: blood, RS: rectal swab, PL: peritoneal liquid, WS: wound swab, BA: bronchial aspirate. IMP: imipenem, IMR: imipenem-relebactam, MEM: meropenem, MEV: meropenem-vaborbactam, AZT: aztreonam, AZA: aztreonam-avibactam. PA-VAP: Pseudomonas aeruginosa ventilator-associated pneumonia. ^#^ The patient received two courses of treatment separated by 40 days.

**Table 2:** Genomic analysis of KPC-producing *K. pneumoniae* clinical isolates.

Patient	Isolate	Therapy	cgMLST	Beta-Lactamases	Other Resistance Determinants	IncFIB(K)	*fec* Operon
1	CHURS_943	Pre	53	*bla*_SHV-11_, *bla*_KPC-3_, *bla*_TEM-1_	*aac(6′)-Ib, aadA2, aph(3′)-Ia, fosA6, mph(A), oqxA, oqxB, sul1, dfrA12, catA1*	Y	Y
	CHURS_966	Post	53	*bla*_SHV-11_, *bla*_KPC-31_, *bla*_TEM-1_	*aac(6′)-Ib, aadA2, aph(3′)-Ia, fosA6, mph(A), oqxA, oqxB, sul1, dfrA12, catA1*	Y	Y
2	CHURS_170942	Pre	3291	*bla*_SHV-11_, *bla*_KPC-3_, *bla*_TEM-1_	*aac(6′)-Ib, aadA2, aph(3′)-Ia, fosA6, mph(A), oqxA, oqxB, sul1, dfrA12, catA1*	Y	Y
	CHURS_171513	Post	3291	*bla*_SHV-11_, *bla*_KPC-94_, *bla*_TEM-1_	*aac(6′)-Ib, aadA2, aph(3′)-Ia, fosA6, mph(A), oqxA, oqxB, sul1, dfrA12, catA1*	Y	Y
3	CHURS_175691	Pre	53	*bla*_SHV-11_, *bla*_KPC-3_, *bla*_TEM-1_	*aac(6′)-Ib, fosA6, oqxA, oqxB*	N	N
	CHURS_175871	Post	53	*bla*_SHV-11_, *bla*_KPC-31_, *bla*_TEM-1_	*aac(6′)-Ib, fosA6, oqxA, oqxB*	N	N
4	CHURS_180130	Pre	3291	*bla*_SHV-11_, *bla*_KPC-3_, *bla*_TEM-1_	*aac(6′)-Ib, fosA6, oqxA, oqxB*	N	N
	CHURS_180510	Post	3291	*bla*_SHV-11_, *bla*_KPC-95_, *bla*_TEM-1_	*aac(6′)-Ib, aadA2, aph(3′)-Ia, fosA6, mph(A), oqxA, oqxB, sul1, dfrA12, catA1*	Y	Y
5	CHURS_182834	Pre	3291	*bla*_SHV-11_, *bla*_KPC-3_, *bla*_TEM-1_	*aac(6′)-Ib, aadA2, aph(3′)-Ia, fosA6, mph(A), oqxA, oqxB, sul1, dfrA12, catA1*	Y	Y
	CHURS_183111	Post	20187	*bla*_SHV-11_, *bla*_KPC-39_, *bla*_TEM-1_	*aac(6′)-Ib, aadA2, aph(3′)-Ia, fosA6, mph(A), oqxA, oqxB, sul1, dfrA12, catA1*	Y	Y
	CHURS_183358	Post	20188	*bla*_SHV-11_, *bla*_KPC-48_, *bla*_TEM-1_	*aac(6′)-Ib, aadA2, aph(3′)-Ia, fosA6, mph(A), oqxA, oqxB, sul1, dfrA12, catA1*	Y	Y
	CHURS_183483	Post	3291	*bla*_SHV-11_, *bla*_KPC-31_, *bla*_TEM-1_	*aac(6′)-Ib, aadA2, aph(3′)-Ia, fosA6, mph(A), oqxA, oqxB, sul1, dfrA12, catA1*	Y	Y
	CHURS_183860	Post	20189	*bla*_SHV-11_, *bla*_KPC-47_, *bla*_TEM-1_	*aac(6′)-Ib, aadA2, aph(3′)-Ia, fosA6, mph(A), oqxA, oqxB, sul1, dfrA12, catA1*	Y	Y
6	CHURS_196071	Pre	3291	*bla*_SHV-11_, *bla*_KPC-3_, *bla*_TEM-1_	*aac(6′)-Ib, aadA2, aph(3′)-Ia, fosA6, mph(A), oqxA, oqxB, sul1, dfrA12, catA1*	Y	Y
	CHURS_196228	Post	3291	*bla*_SHV-11_, *bla*_KPC-148_, *bla*_TEM-1_	*fosA, oqxA, oqxB, catA1*	Y	N
7	CHURS_196061	Pre	5128	*bla*_SHV-11_, *bla*_KPC-3_, *bla*_TEM-1_	*aac(6′)-Ib, aadA2, aph(3′)-Ia, fosA6, mph(A), oqxA, oqxB, sul1, dfrA12, catA1*	Y	Y
	CHURS_196226	Post	5128	*bla*_SHV-11_, *bla*_KPC-31_, *bla*_TEM-1_	*aac(6′)-Ib, aadA2, aph(3′)-Ia, fosA6, mph(A), oqxA, oqxB, sul1, dfrA12, catA1*	Y	Y
8	CHURS_201901	Pre	5128	*bla*_SHV-11_, *bla*_KPC-3_, *bla*_TEM-1_	*aac(6′)-Ib, aadA2, aph(3′)-Ia, fosA6, mph(A), oqxA, oqxB, sul1, dfrA12, catA1*	Y	Y
	CHURS_202826	Post	5128	*bla*_SHV-11_, *bla*_KPC-85_, *bla*_TEM-1_	*aac(6′)-Ib, aadA2, aph(3′)-Ia, fosA6, mph(A), oqxA, oqxB, sul1, dfrA12, catA1*	Y	Y
9	CHURS_231184	Pre	3291	*bla*_SHV-11_, *bla*_KPC-3_, *bla*_TEM-1_	*aac(6′)-Ib, fosA6, oqxA, oqxB*	N	N
	CHURS_231595	Post	21209	*bla*_SHV-11_, *bla*_KPC-178_, *bla*_TEM-1_	*aac(6′)-Ib, fosA6, oqxA, oqxB*	N	N
	CHURS_231596	Post	3291	*bla*_SHV-11_, *bla*_KPC-28_, *bla*_TEM-1_	*aac(6′)-Ib, fosA6, oqxA, oqxB*	N	N

**Table 3:** MICs of CAZ, CAZ/AVI and FDC (two assays) determined against *E. coli*-TOP10 containing different KPC variants.

Strain	Amino Acid Change	Localization in the Protein	MIC (mg/L)			
			CAZ	CAZ/AVI	FDC 1	FDC 2
*E coli* TOP10	NA	NA	0.125	0.125	≤0.03	≤0.03
*E. coli* p (KPC-3)	NA	NA	>256	0.75	0.06	0.06
*E. coli* p (KPC-28)	del_242-243_GT	Loop 237–243	>256	>256	2	2
*E. coli* p (KPC-31)	D179Y	Ω-loop	>256	>256	1	2
*E. coli* p (KPC-39)	A172D	Ω-loop	64	8	0.06	0.06
*E. coli* p (KPC-47)	A172T + T243A	Ω-loop + loop 237–243	>256	16	0.25	0.25
*E. coli* p (KPC-48)	L169P + A172T	Ω-loop	24	2	0.125	0.06
*E. coli* p (KPC-85)	A172V	Ω-loop	96	3	0.06	0.06
*E. coli* p (KPC-94)	L169H + N170del	Ω-loop	>256	8	0.5	0.5
*E. coli* p (KPC-95)	A172T + D179Y	Ω-loop	>256	>256	4	4
*E. coli* p (KPC-148)	Ins275_EAVYTRAPNKDDKYS	Loop 266–275	256	32	0.25	0.25
*E. coli* p (KPC-178)	P174L	Ω-loop	128	16	0.125	0.06

NA: Not applicable.

## Data Availability

Sequence data have been deposited in NCBI under BioProject number PRJNA1474786.

## Supplementary Materials

The following supporting information can be downloaded at: https://www.mdpi.com/article/10.3390/antibiotics15070701/s1, Figure S1: Phylogenetic tree; Table S1: Variant call results; Table S2: cgSNP distance matrix.
