# Supplementary material for: Emerging Cross-Resistance to Cefiderocol and Ceftazidime-Avibactam in KPC-Producing Klebsiella pneumoniae During Ceftazidime-Avibactam Therapy
Source: Antibiotics (Basel). 2026 Jul 17;15(7):701. doi: 10.3390/antibiotics15070701 (PMC13405771; doi:10.3390/antibiotics15070701)
Supplement: Supplementary file 1 [file antibiotics-15-00701-s001.zip › Table S2 Figure S1.pdf]

Table S2. cgSNP distance matrix among the isolates.

| | CHURS 943 | CHURS 966 | CHURS 170942 | CHURS 171513 | CHURS 175691 | CHURS 175871 | CHURS 180130 | CHURS 180510 | CHURS 182834 | CHURS 183111 | CHURS 183358 | CHURS 183483 | CHURS 183860 | CHURS 196071 | CHURS 196228 | CHURS 196061 | CHURS 196226 | CHURS 201901 | CHURS 202826 | CHURS 231184 | CHURS 231595 | CHURS 231596 |
|---|---|---|---|---|---|---|---|---|---|---|---|---|---|---|---|---|---|---|---|---|---|---|
| CHURS 943 | 0 | 25 | 40 | 42 | 24 | 26 | 27 | 23 | 28 | 49 | 73 | 31 | 123 | 28 | 35 | 46 | 49 | 49 | 49 | 33 | 37 | 38 |
| CHURS 966 | 25 | 0 | 37 | 39 | 25 | 25 | 14 | 8 | 15 | 36 | 60 | 16 | 110 | 15 | 22 | 43 | 44 | 46 | 46 | 20 | 24 | 25 |
| CHURS 170942 | 40 | 37 | 0 | 2 | 40 | 42 | 39 | 35 | 40 | 61 | 85 | 43 | 135 | 40 | 47 | 52 | 55 | 55 | 55 | 45 | 49 | 50 |
| CHURS 171513 | 42 | 39 | 2 | 0 | 42 | 42 | 41 | 37 | 42 | 63 | 87 | 45 | 137 | 42 | 49 | 54 | 57 | 57 | 57 | 47 | 51 | 52 |
| CHURS 175691 | 24 | 25 | 40 | 42 | 0 | 2 | 27 | 23 | 28 | 49 | 73 | 31 | 123 | 28 | 35 | 46 | 49 | 49 | 49 | 33 | 37 | 38 |
| CHURS 175871 | 26 | 25 | 42 | 42 | 2 | 0 | 29 | 23 | 30 | 51 | 75 | 31 | 125 | 30 | 37 | 48 | 49 | 51 | 51 | 35 | 39 | 40 |
| CHURS 180130 | 27 | 14 | 39 | 41 | 27 | 29 | 0 | 8 | 17 | 38 | 62 | 20 | 112 | 17 | 24 | 45 | 48 | 48 | 48 | 22 | 26 | 27 |
| CHURS 180510 | 23 | 8 | 35 | 37 | 23 | 23 | 8 | 0 | 11 | 32 | 56 | 12 | 106 | 11 | 18 | 41 | 42 | 44 | 44 | 16 | 20 | 21 |
| CHURS 182834 | 28 | 15 | 40 | 42 | 28 | 30 | 17 | 11 | 0 | 21 | 45 | 3 | 95 | 8 | 15 | 46 | 49 | 49 | 49 | 13 | 17 | 18 |
| CHURS 183111 | 49 | 36 | 61 | 63 | 49 | 51 | 38 | 32 | 21 | 0 | 36 | 24 | 86 | 29 | 36 | 67 | 70 | 70 | 70 | 34 | 38 | 39 |
| CHURS 183358 | 73 | 60 | 85 | 87 | 73 | 75 | 62 | 56 | 45 | 36 | 0 | 48 | 104 | 53 | 60 | 91 | 94 | 94 | 94 | 58 | 62 | 63 |
| CHURS 183483 | 31 | 16 | 43 | 45 | 31 | 31 | 20 | 12 | 3 | 24 | 48 | 0 | 98 | 11 | 18 | 49 | 50 | 52 | 52 | 16 | 20 | 21 |
| CHURS 183860 | 123 | 110 | 135 | 137 | 123 | 125 | 112 | 106 | 95 | 86 | 104 | 98 | 0 | 103 | 110 | 141 | 144 | 144 | 144 | 108 | 112 | 113 |
| CHURS 196071 | 28 | 15 | 40 | 42 | 28 | 30 | 17 | 11 | 8 | 29 | 53 | 11 | 103 | 0 | 7 | 46 | 49 | 49 | 49 | 13 | 17 | 18 |
| CHURS 196228 | 35 | 22 | 47 | 49 | 35 | 37 | 24 | 18 | 15 | 36 | 60 | 18 | 110 | 7 | 0 | 53 | 56 | 56 | 56 | 20 | 24 | 25 |
| CHURS 196061 | 46 | 43 | 52 | 54 | 46 | 48 | 45 | 41 | 46 | 67 | 91 | 49 | 141 | 46 | 53 | 0 | 3 | 11 | 11 | 51 | 55 | 56 |
| CHURS 196226 | 49 | 44 | 55 | 57 | 49 | 49 | 48 | 42 | 49 | 70 | 94 | 50 | 144 | 49 | 56 | 3 | 0 | 14 | 14 | 54 | 58 | 59 |
| CHURS 201901 | 49 | 46 | 55 | 57 | 49 | 51 | 48 | 44 | 49 | 70 | 94 | 52 | 144 | 49 | 56 | 11 | 14 | 0 | 0 | 54 | 58 | 59 |
| CHURS 202826 | 49 | 46 | 55 | 57 | 49 | 51 | 48 | 44 | 49 | 70 | 94 | 52 | 144 | 49 | 56 | 11 | 14 | 0 | 0 | 54 | 58 | 59 |
| CHURS 231184 | 33 | 20 | 45 | 47 | 33 | 35 | 22 | 16 | 13 | 34 | 58 | 16 | 108 | 13 | 20 | 51 | 54 | 54 | 54 | 0 | 4 | 5 |
| CHURS 231595 | 37 | 24 | 49 | 51 | 37 | 39 | 26 | 20 | 17 | 38 | 62 | 20 | 112 | 17 | 24 | 55 | 58 | 58 | 58 | 4 | 0 | 9 |
| CHURS 231596 | 38 | 25 | 50 | 52 | 38 | 40 | 27 | 21 | 18 | 39 | 63 | 21 | 113 | 18 | 25 | 56 | 59 | 59 | 59 | 5 | 9 | 0 |

https://doi.org/10.3390/antibiotics15070701

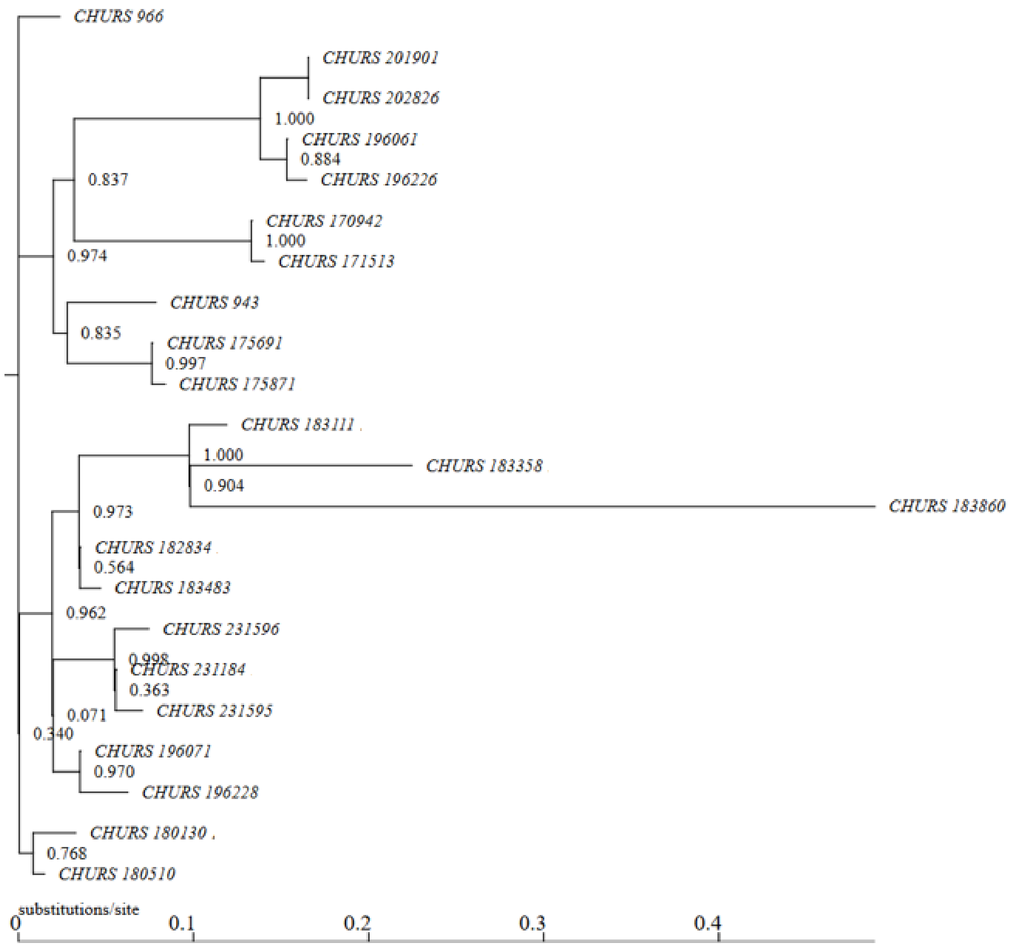


Figure S1. Phylogenetic tree obtained using CSIPhylogeny comprising all the 22 genomes from this study. The tree was generated with the distance method algorithm. Branch lengths are proportional to the number of evolutionary events.
